# Antiphospholipid antibodies in healthy Serbian middle-aged subjects: Preliminary data

**DOI:** 10.5937/jomb0-35642

**Published:** 2022-10-15

**Authors:** Mirjana B. Bećarević, Snežana Jovičić, Svetlana D. Ignjatović, Duško Mirković

**Affiliations:** 1 University of Novi Sad, Faculty of Medicine, Department of Pharmacy, Novi Sad; 2 University of Belgrade, Faculty of Pharmacy, Department of Medical Biochemistry, Belgrade; 3 Clinical University Center of Serbia, Center for Medical Biochemistry, Belgrade

**Keywords:** antiphospholipid antibodies, apolipoproteins, complement components, C-reactive protein, haptoglobin, serum amylod A, antifosfolipidn antitela, apolipoproteini, komplement komponente, C-reaktivni protein, haptoglobin, serum amilod A

## Abstract

**Background:**

The investigation of the prevalence of the IgG and the IgM isotypes of anticardiolipin (aCL) and antib2glycoprotein I (ab2gpI) Abs in healthy Serbian middleaged subjects was the main goal of our study. In addition, we analyzed the potential associations of above-mentioned Abs with serum proteins and lipids/lipoproteins.

**Methods:**

Forty healthy subjects were included in our study. Obesity (BMI 30 kg/m2) was present in 8/40 (20%) subjects. Titers of analyzed Abs were measured by ELISA.

**Results:**

The prevalence of IgG and IgM ab2gpI Abs was 5% and 12.5%, respectively, while the prevalence of IgM aCL was 10%. The IgG ab2gpI Abs were significantly different between subjects with normal triglycerides levels and those with hypertriglyceridemia (Mann-Whitney, P = 0.014). The significant difference in hsCRP concentrations was observed between subjects with the increased levels of the IgM isotype of aCL Abs and those with normal IgM aCL values (Mann-Whitney, P = 0.028).

**Conclusions:**

Dyslipidemia and BMI ≥30 were associated with aPL Abs and therefore, the correction of BMI and lipid status might be beneficial in reduction or elimination of predisposing factors that might trigger thrombotic events in otherwise healthy middle-aged subjects. Larger national study is necessary to confirm our findings.

## Abstract (sr)

**Uvod:**

Analiza prevalentnosti IgG i IgM izotipa antikardiolipinskih (aCL) i anti-b2glikoprotein I (ab2gpI) At kod zdravih sredovečnih stanovnika Srbije je bila glavni cilj naše studije. Dodatno, analizirali smo potencijalnu povezanost gore-navedenih At sa serumskim proteinima i lipidima/lipoproteinima.

**Metode:**

40 zdravih ispitanika je bilo uključeno u našu studiju. Gojaznost (BMI ≥ 30 kg/m 2) je uočena kod 8/40 (20%) osoba. Titri analiziranih antitela su utvrđivani ELISA testom.

**Rezultati:**

Prevalentnost IgG i IgM ab2gpIAt je bila 5% i 12.5%, redom, dok je prevalentnost IgM aCL bila 10%. Nivoi IgG ab2gpI At su se značajno razlikovali izmenu ispitanika sa i bez hipertrigliceridemije (Mann-Whitney, P = 0.014). Značajne razlike u hsCRP koncentracijama uočene su izmenu osoba sa povišenim nivoima IgM aCL At i onih sa referentnim vrednostima (Mann-Whitney, P = 0,028).

**Zaključak:**

Dislipidemija i BMI ≥30 su bili povezani sa aPL At uprkos njihovoj niskoj prevalentnosti, i zato korekcija BMI i lipidnog statusa bi bila korisna u redukciji ili eliminaciji predispozirajućih faktora koji mogu da izazovu trombotički događaj kod inače zdravih sredovečnih ispitanika. Obimnije nacionalne studije su neophodne da bi potvrdile naše nalaze.

## Introduction

Antiphospholipid antibodies (aPL Abs) represent a diverse group of Abs directed against complexes formed between negatively charged phospholipids (i.e. cardiolipin (CL), phosphatidyl-serine, phosphatidyl-inositol etc.) or blood proteins (i.e. beta 2 glycoprotein I (β2gpI)), etc [1] [2] [3] [4] [5].

Increased titers of aPL Abs are the main laboratory feature of the antiphospholipid syndrome (APS). It is an autoimmune disease that is (beside the presence of aPL Abs) characterized by the presence of recurrent thrombosis and/or pregnancy losses [6] [7]. According to the latest classification criteria for the diagnosis of the APS [8] [9], the presence of aPL Abs (i.e. the IgG and/or the IgM isotype of the anticardiolipin (aCL) and/or the IgG and/or the IgM isotype of the anti-β2 glycoprotein I (β2gpI) Abs) must be present at medium to high titers in two or more occasions, at least twelve weeks apart. The reasons why β2gpI and CL, as ubiquitously present autoantigens in some persons promotes production of pathogenic autoAbs remains elusive.

There are several studies that investigated the prevalence of aPL Abs in healthy subjects of various nationalities [10] [11] [12] and numerous studies that compared levels of aPL Abs in patients with different autoimmune disease vs. healthy subjects [13] [14] [15] [16]. We have previously reported [13] that in 47 Serbian young (mean ± SD, 39.68 ± 13.93, 33 female) lean, healthy adults, levels of aPL Abs were significantly lower in comparison to patients with primary APS and that levels of analyzed serum lipids (cholesterol, HDL-cholesterol, LDL-cholesterol and triglycerides) were below cut-off values in these young subjects. However, no studies that analyze the association of aPL Abs with sera lipids/lipoproteins and proteins in healthy Serbian middle-aged subjects are available. Therefore, the aim of our study was to evaluate the prevalence of aPL Abs in healthy middle-aged Serbian subjects and to investigate the potential correlation between aPL Abs positivity and lipids/lipoproteins (cholesterol, HDL-cholesterol, LDL-cholesterol, triglycerides, apolipoproteins (apo) AI, B and lipoprotein (a) (Lp(a)) and serum proteins (C-reactive protein (CRP), serum amyloid A (SAA), haptogblobin (HPT), fibrinogen, C3 and C4 complement components).

## Materials and methods

### Subjects

All procedures performed in our study were in accordance with Helsinki declaration (and its later amendments) and with the ethical standards of the institutional ethical committee. Written informed consent was obtained from all individual participants included in the study.

Our study included 40 healthy subjects (mean age ± SD, 58.22 ± 3.47) comprised from our colleagues and our acquaintances that did not show any clinical signs of thrombosis, pregnancy morbidity, infections, cancer and autoimmune diseases. The use of the laboratory information system data for this study was approved by our local Ethical Committee (Ethical Committee of the Clinical University Center of Serbia, Approval No 4815/3). Female to male ratio was 16/24. The body mass index (BMI) was calculated as the weight (kg)/height^2^ (m^2^).

### Methods

Serum concentrations of total cholesterol (TC), HDL-cholesterol (HDL-C), LDL-cholesterol (LDL-C), triglycerides (TG) and high sensitivity C-reactive protein (hsCRP) were measured on Olympus AU2700 automated analyzer (Beckman Coulter Inc, USA). Total cholesterol and TG concentrations were determined using standard enzymatic assays. High-density lipoprotein cholesterol concentrations were determined using direct enzymatic method. Friedwald formula was used to calculate LDL-C concentrations, but if TG concentrations >4.50 mmol/L direct enzymatic method was used.

Concentrations of hsCRP were measured with an immunoturbidimetric latex assay. Cut-off values for TC, HDL-C, LDL-C, TG and hsCRP were 5.0 mmol/L, 1.55 mmol/L, 2.5 mmol/L, 1.70 mmol/L, 3 mg/L (for high cardiovascular risk), respectively.

Apolipoprotein A-I, apoB, Lp(a), C3 and C4 complement components (C3, C4) were determined by immunoturbidimetric procedures on Architect c8000 chemistry system (Abbott Laboratories, Illinois, USA). Reference ranges for apoAI and apoB were: 0.95-1.86 g/L (men), 1.01-2.23 g/L (women); 0.49-1.73 g/L (men), 0.53-1.82 g/L (women), respectively. Cut-off value for Lp(a) was 300 mg/L. Reference ranges for C3 and C4 complement components were: 0.82-1.85 g/L (men), 0.83-1.93 g/L (women); 0.15-0.53 g/L (men), 0.15-0.57 g/L (women), respectively [17].

Serum concentration of SAA and HPT were assayed using particle-enhanced immunonephelometry with BN II nephelometer (Siemens Healthcare GmbH, Germany). Cut-off value for SAA was 6.4 mg/L, while the reference range for HPT was 0.3-2 g/L. Fibrinogen concentrations were measured in citrate plasma by prothrombin time (PT)-based method on ACL 7000 analyzer (Instrumentation Laboratory SpA, Milan, Italy). For each analyzer appropriate supplied reagents were used. Reference range for fibrinogen was 1.7-5.4 g/L.

Antibody levels were estimated by ELISA in patient sera using commercially available reagents of ORGENTEC, Diagnostika GmbH, Germany for the detection of anti-β2gpI (IgG and IgM isotypes) and anticardiolipin (aCL) (IgG and IgM isotypes) antibodies. Cut-off values were set in accordance to manufacturer recommendation (8 U/mL (for the IgG and IgM isotypes of aβ2gpI Abs), 10 GPL-U/mL (for IgG aCL) and 7 MPLU/mL (for IgM aCL Abs).

### Statistical analysis

Shapiro-Wilik test was used to study whether analyzed variables followed a normal distribution. The categoric variables were expressed in percentages (%), while continuous variables were expressed as mean ± SD in the case of normal distribution, but if concentrations did not follow a normal distribution pattern, the values were expressed as median (25th - 75th percentiles). Mann-Whitney test, Kruskal-Wallis and 2-test were used, when appropriate. The correlation between two quantitative variables was determined with the Spearman`s correlation test. In all of the above-mentioned tests, *P* < 0.05 was considered statistically significant. Analyses were conducted in SPSS 20 (SPSS, Inc, Chicago, IL, USA).

## Results

Obesity (BMI ≥ 30 kg/m^2^) was present in 2/16 (12.5%) female subjects and in 6/24 (25%) male subjects. Increased BMI values (BMI 25-30 kg/m^2^) were present in eight (50%) female and in eight (33.33%) male subjects. Our study included 5/16 (31.25%) female smokers and 6/24 (25%) male smokers.

Serologic features of analyzed subjects are presented in Table 1. Not a single subject had increased titers of the IgG isotype of aCL Abs. Increased levels of the IgM isotype of aCL Abs were present in 4 (2 female and 2 male)/40 (10%) of analyzed subjects. Elevated levels of the IgG isotype of ab2gpI Abs were present in only two male subjects (5%), while increased IgM ab2gpI levels were observed in 5 (4 female and 1 male)/40 (12.5%) of analyzed subjects. Simultaneous presence of the IgM isotype of both aCL and ab2gpI Abs was present in three subjects (7.5%).

**Table 1:** Concentrations analyzed parameters in female and male subjects (comparison was done by Mann-Whitney, *P < 0.05).

Parameters Median (25th–75th)	Femalen = 16	Malen = 24	P value
Age (years, (mean ± SD))	57.19 ± 3.31	58.92 ± 3.48	0.130
BMI (kg/m^2^)	26.34 (24.33 – 28.86)	27.44 (23.77 – 30.04)	0.782
Glucose (mmol/L)	5.25 (5.02 – 6.02)	5.45 (5.20 – 5.87)	0.589
Cholesterol (mmol/L)	6.23 (5.80 – 7.60)	6.15 (5.69 – 6.77)	0.464
Triglycerides (mmol/L)	1.63 (1.36 – 2.39)	1.57 (1.06 – 2.61)	0.879
LDL–cholesterol (mmol/L)	4.23 (3.71 – 4.84)	3.78 (3.15 – 4.30)	0.100
HDL-cholesterol (mmol/L)	1.30 (1.15 – 1.71)	1.28 (1.05 – 1.48)	0.499
ApoAI (g/L)	1.58 (1.47 – 1.75)	1.56 (1.38 – 1.77)	0.629
ApoB (g/L)	1.24 (1.09 – 1.44)	1.25 (1.05 – 1.42)	0.782
Lp(a) (g/L)	0.17 (0.08 – 0.55)	0.16 (0.03 – 0.32)	0.362
CRP (mg/L)	2.05 (1.29 – 3.76)	1.54 (1.08 – 3.37)	0.508
SAA (mg/L)	4.55 (2.22 – 9.05)	3.35 (1.45 – 6.85)	0.334
Fibrinogen (g/L)	3.91 (2.97 – 4.50)	4.22 (3.8 – 4.47)	0.163
C3 (g/L)	1.43 (1.30 – 1.51)	1.51 (1.13 – 1.63)	0.659
C4 (g/L)	0.27 (0.24 – 0.32)	0.26 (0.21 – 0.30)	0.415
HPT (g/L)	1.39 (0.98 – 1.67)	1.21 (0.98 – 1.72)	0.730
aCL IgG (GPL U/mL)	3.29 (1.89 – 4.33)	2.84 (1.59 – 3.55)	0.282
aCLIgM (MPL U/mL)*	2.76 (1.79 – 5.07)	2.03 (1.33 – 2.78)	0.034*
ab2gpI IgG (U/mL)	2.54 (1.77 – 3.44)	2.16 (1.66 – 2.77)	0.281
ab2gpI IgM (U/mL)*	3.63 (2.19 – 7.86)	2.19 (1.47 – 3.55)	0.011*

**Figure 1:**
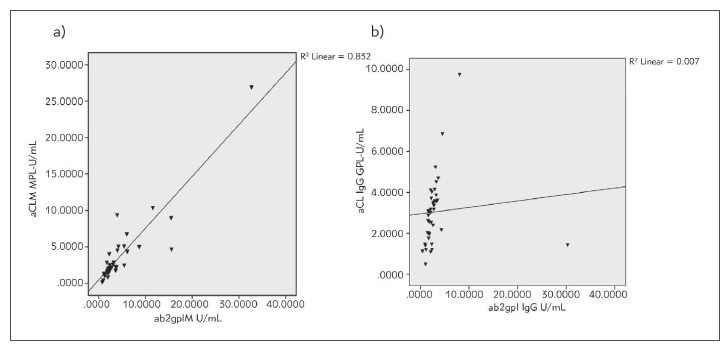
Correlation between the IgM (r = 0.882, P = 0.000, (Panel (a) and the IgG (r = 0.632, P = 0.000, (Panel (b) isotypes of anticardiolipin (aCL) and anti-b2glycoprotein I (ab2gpI) antibodies

The IgM isotype of aCL and aβ2gpI Abs were in positive correlation (r = 0.882, *P *= 0.000) (Figure 1, *Panel A*). The IgM class of aCL Abs and the IgG isotype of aβ2gpI Abs were in positive correlation (r = 0.319, *P* = 0.045). A positive correlation was found for the IgG and the IgM isotype of aCL Abs (r = 0.502, *P* = 0.001). In addition, the IgG isotype of aCL Abs was in positive correlation with both the IgG (r = 0.632, *P* = 0.000) , Figure 1, *Panel B*) and the IgM (r = 0.421, *P* = 0.007) isotypes of aβ2gpI antibodies.

The hypertriglyceridemia was noticed in 17/40 (42.5%) of analyzed subjects and the concentrations of the IgG isotype of aβ2gpI Abs were significantly different between subjects with and without hypertriglyceridemia (Mann-Whitney, *P *= 0.014). Subjects with increased Lp(a) levels and those with normal Lp(a) values had significantly different IgG aβ2gpI Abs concentrations (Mann-Whitney, *P* = 0.026). However, only male subjects showed a positive correlation between Lpa(a) and the IgM isotype of aβ2gpI Abs (r = 0.412, *P* = 0.045).

Kruskal-Wallis test (One-way ANOVA) revealed that the IgG isotype of aβ2gpI Abs (*P* = 0.020), hsCRP (*P* = 0.048), C3c (*P *= 0.015), cholesterol (P= 0.028) and triglycerides (P= 0.012) concentrations were significantly different among subjects with different BMI values (obese, increased and normal BMI). The significant difference in body weight values was observed between subgroups of subjects with increased IgM isotype of aβ2gpI Abs and those without it (i.e. normal IgM aβ2gpI concentrations, Mann-Whitney, *P* = 0.034), while significant difference in hsCRP concentrations was observed between subjects with the increased levels of the IgM isotype of aCL Abs and those with normal IgM aCL values (Mann-Whitney, *P* = 0.028).

No correlation between the analyzed Abs and C3c and C4 complement components, SAA, CRP, HPT, fibrinogen and apolipoproteins was obtained. However, hsCRP was in positive correlation with SAA (r = 0.511, *P* = 0.001), C3c (r = 0.469, *P* = 0.002), C4 (r = 0.377, *P* = 0.017) and HPT (r = 0.388, *P* = 0.013). Fibrinogen concentrations were in positive correlation with triglycerides (r = 0.332, *P* = 0.037) and C3c (r = 0.479, *P* = 0.002).

## Discussion

McIntyre et al. [12] have published that 63/775 (8.1%) volunteer blood donors (average age: 43 years, range) had positive finding of one or more aPL Abs [12]. Another study [18] has revealed that 20.7% of centenarians were positive for IgG aCL and 2.59% for IgM aCL Abs, while 54.3% of centenarians were positive for the IgG aβ2gpI and 8.6% for IgM aβ2gpI Abs [18]. Despite this high aPL Abs prevalence in centenarians (comparable to titers observed in APS patients), the authors of the study did not observe a single vascular event and therefore the authors have suggested that some »unknown protective factor and/or lacking of triggering factors« are responsible for their findings [18]. Similarly, Mustonen et al. [19] have reported that single aPL Abs positivity does not seem to carry an elevated risk of thrombosis (in asymptomatic aPL Abs carriers). Avčin et al. [20] reported no statistically significant differences in the frequency of the elevated either aCL isotype between blood donors (mean age: 34 years, range (18-65)) and analyzed children (preschool and adolescent), i.e. 5/52 (9.6%) blood donors were positive for aCL Abs (5.8% were positive for IgG aCL Abs vs. 7/61 (11.4%) of analyzed children were positive for IgG aCL). The same group [20] have reported no differences in the frequency of the either isotype of aβ2gpI Abs between blood donors and analyzed children (4/52 (7.7%) of blood donors were positive for aβ2gpI Abs and 1/52 (1.9%) were positive for IgG aβ2gpI Abs) vs. 4/61 (6.6%) of analyzed children and (2/61 (3.3% were positive for IgG aB2gpI Abs)). However, Avčin et al. [20] did not analyze the gender differences in regard with the prevalence of aPL Abs in blood donors and children. McIntyre et al. [12] have reported that males were positive for aCL more often than females, but no differences in aPL isotypes between gender was observed [12]. In addition, the same authors [12] have reported the persistence of the IgM aCL Abs (in a healthy male repeat blood donor (age: 60 years)) even after 16 months interval between blood draws. Although one might expect the isotype switch from IgM to IgG to occur with the loss of the IgM isotype in the normal antibody response, the authors [12] did not provide an explanation for their observation.

In comparison to above-mentioned studies, our study population was comprised from middle-aged subjects (mean age ± SD, 58.22 ± 3.47) and we observed that not a single subject had increased titers of the IgG isotype of aCL Abs, but elevated IgM aCL Abs levels were present in 10% of analyzed persons, while increased titers of the IgG and IgM aβ2gpI were observed in 5% and 12.5% of analyzed subjects, respectively. We have observed that the IgM isotype was more frequent than the IgG isotype but this was not statistically significant.

Previously it was reported that β2gpI (also known as apolipoprotein (apo) H) was implicated in atherogenic and thrombotic processes and that serum β2gpI concentrations were elevated in patients with primary hyperlipidaemia [21]. In addition, significant correlations were observed between β2gpI and triglycerides and total cholesterol concentrations [21]. Although in our study we did not measure serum β2gpI levels, we observed that IgG aβ2gpI Abs concentrations were significantly different between subjects with and without hypertriglyceridemia. In addition, the concentrations of IgG aβ2gpI Abs were significantly different between subjects with increased Lp(a) levels and those with normal levels. Interestingly, only analyzed male subjects showed a positive correlation between the IgM aβ2gpI Abs and Lp(a) concentrations.

Previously it was reported that concentrations of IgG and IgM aCL Abs were similar in obese and nonobese patients with primary APS [22]. In agreement, no differences in regard to either isotype of aCL Abs were observed in our subjects considering their BMI values. However, in our group, obesity was present in 12.5% of females and in 25% of males and we noticed that subjects with different BMI values had significantly different concentrations of the IgG aβ2gpI Abs, hsCRP and C3c. Elevated hsCRP levels and the presence of aPL Abs exhibited some similarities in the pathogenesis of thrombosis [23]. It is considered that hsCRP is a predictor of vascular events independently of all other lipids and non-lipid risk factors [23]. Lin et al. [24] have reported that in patients with inflammation, β2gpI levels were in negative correlation with CRP and in positive correlation with negative acute phase proteins (such as albumin and transferrin). It was reported that acute phase proteins (such as SAA) have been associated with the pathology of anti-β2gpI Abs and that SAA levels were increased and correlated with the history of thrombosis in APS patients [25], while in healthy young Japanese, no correlation between CRP and SAA levels was observed [26]. In our study, hsCRP concentrations were significantly different between subjects with increased IgM aCL Abs titers and those without it. In addition, we observed significant correlation between hsCRP and SAA, HPT and complement components (C3c and C4). In patients with idiopathic aPL Abs, fibrinogen concentrations correlated with the aCL IgG Abs and the authors [27] suggested that measurement of fibrinogen may be beneficial in defining aPL subjects with higher thrombotic risk that might require pharmacological intervention for lowering fibrinogen levels [27]. In our study, fibrinogen concentrations did not correlate with the either isotype of analyzed Abs.

In conclusion, BMI 30 (obesity) and dyslipidemia were associated with aPL Abs despite their low prevalence in analyzed subjects. Antiphospholipid antibodies are regarded as natural autoantibodies and due to »molecular mimicry between microbial epitopes and human β2gpI it is possible that in genetically predisposed subjects, generation of aPL Abs might be initiated« [28]. However, predisposing factors are not completely elucidated yet and there are several reports that suggest that pro-inflammatory cytokines and acute phase reactants [25] are important for generating »second hit« that is vital in the pathology of aPL Abs. Our study provides a rationale for the fact that correction of BMI and lipid status might be beneficial in reduction and/or elimination of predisposing factors that might trigger thrombotic events in otherwise healthy Serbian middle-aged subjects. In addition, our results should be regarded with caution (i.e. as preliminary) due to relatively small number of participants included in the study and therefore, larger national study is necessary to confirm our findings.

## Dodatak

### List of abbreviations

Abs, antibodies;<br>aβ2gpI, anti-b2glycoprotein I Abs;<br>aCL, anticardiolipin Abs;<br>aPL, antiphospholipid Abs;<br>APS, antiphospholipid syndrome.

### Acknowledgment

The authors of the article have no conflict of interest (financial nor non-financial) related to this manuscript. All authors approved the final manuscript as submitted and agree to be accountable for all aspects of the work (MB is responsible for analysis, interpretation of data and writing of the Article; SJ and MB were responsible for laboratory measurements of investigated parameters; SJ was responsible for recruitment of subject and providing their data; SI and DM are responsible for the final approval of the Article).

The present work was supported by the Ministry of Science, Education and Technological Development of the Republic of Serbia on the basis of contracts No.175036 and No.451-03-68/2020-14/200161.

### Conflict of interest statement

All the authors declare that they have no conflict of interest in this work.
